# Detection of *Tuber melanosporum* Using Optoelectronic Technology

**DOI:** 10.3390/s26010230

**Published:** 2025-12-30

**Authors:** Sheila Sánchez-Artero, Antonio Soriano-Asensi, Pedro Amorós, Jose Vicente Ros-Lis

**Affiliations:** 1 Instituto Interuniversitario de Investigación de Reconocimiento Molecular y Desarrollo Tecnológico (IDM), Universitat Politècnica de València, Universitat de València, Doctor Moliner 50, 46100 Burjassot, Valencia, Spain; 2 REDOLí Research Group, Universitat de València, Doctor Moliner 50, 46100 Burjassot, Valencia, Spain; 3 Departament d’Informàtica, ETSE, Universitat de València, Av de la Universidad s/n, 46100 Burjassot, Valencia, Spain; 4 Institut de Ciència dels Materials (ICMUV), C/Catedrático José Beltrán 2, 46980 Paterna, Valencia, Spain

**Keywords:** optoelectronic nose, *Tuber melanosporum*, partial least squares discriminant analysis (PLS-DA), artificial neural network (ANN)

## Abstract

*Tuber melanosporum*, the black truffle, is a fungus of high economic and ecological value, but its underground detection remains a challenge due to the lack of reliable, non-invasive methods. This study presents the development and proof of concept of a portable optoelectronic nose that integrates nine optical sensors and one electrochemical sensor for the in vitro identification of *T. melanosporum*. The optical sensors use colorimetric and fluorogenic molecular indicators supported on UVM-7, alumina, and silica. Tests were performed with truffles at different depths and in the presence of soil and compost to evaluate the device’s multi-source response. Partial least squares discriminant analysis (PLS-DA) models showed robust discrimination between soil, compost, and truffles, with an accuracy of 0.91 under most conditions. Detection at 30 cm showed an accuracy of 0.94, confirming the system’s ability to differentiate between sample types. Performance improved in simplified scenarios based on the presence or absence of truffles. Furthermore, the artificial neural network models achieved optimal results in binary classification. Taken together, the results support the system’s potential as an accurate, non-invasive tool with possible application to the agronomic management of truffle orchards.

## 1. Introduction

Truffle is a hypogeous fungus that grows in symbiosis with the roots of various plant species, establishing ectomycorrhizal associations that are essential for its development. It is a gourmet product highly prized around the world for its intense, characteristic aroma. More than a hundred species of truffles have been described, belonging mainly to the genera Tuber and Terfezia, although only a few are of significant commercial interest [1]. Among these, *T. melanosporum* stands out for the great complexity of its aromatic profile, composed of a wide variety of volatile compounds. Sensory characteristics include an earthy, musty flavor accompanied by a pungent aroma with slight notes of radish and hints of hazelnut [2].

The high economic value of truffles is due not only to their rarity and unmistakable aromatic bouquet, but also to their nutritional and functional properties. Several studies have pointed out that truffles have biological activities with potential therapeutic interest, including antioxidant, anti-inflammatory, antiviral, hepatoprotective, antimutagenic, immunomodulatory, antitubercular, antitumor, and antimicrobial effects [3]. These attributes confirm *T. melanosporum* as a product of great gastronomic and scientific interest.

The detection of *Tuber melanosporum* through its volatile organic compounds (VOCs) has generated considerable interest in research, as the aromatic profile is a key marker for its identification and authentication. Among the most widely used analytical techniques is gas chromatography coupled with mass spectrometry (GC-MS), which provides a detailed profile of the volatiles, differentiating truffles according to geographical origin or seasonality [3,4]. Complementary methods, such as headspace solid-phase microextraction (HS-SPME) applied to species such as *Tuber aestivum* and *Tuber borchii*, have proven effective in mapping the volatile metabolome of truffles from different sources [5]. In addition, advanced microextraction techniques have been developed for the preconcentration of trace-level VOCs, such as SPME Arrow, multisorbent adsorbent tubes (Tenax TA, Carbopack), and passive in situ sampling devices, which allow for the timed accumulation of analytes and significantly improve sensitivity in complex matrices such as soil [6]. Similarly, simultaneous distillation–extraction has been applied specifically to *T. melanosporum*, confirming its potential for recovering active aromatic compounds [7]. Beyond chromatographic approaches, sensory evaluations supported by chemometric tools such as principal component analysis (PCA) have been implemented, allowing for the identification of key VOCs responsible for the characteristic aroma of *T. melanosporum* [8]. This integrated approach not only facilitates the discrimination between truffle species but also helps prevent adulteration and guarantee product authenticity. Molecular tools have proven to be a robust alternative for identifying *T. melanosporum*, especially in the agro-industrial context [9,10]. Nevertheless, these approaches, although effective, have a high cost and require expensive instrumentation, qualified personnel, and/or are time consuming.

Thus, rapid and non-invasive detection devices have been explored, such as electronic noses, designed to mimic olfactory detection [11]. Among the most widely used sensors are those based on metal oxides (MOSs) [12,13] and quartz microbalance (QMB) [14,15]. Electronic noses have been widely applied both for sample discrimination and for the classification and grading of aromatic products based on their volatile organic compound (VOC) profiles. In truffle analysis, several studies have demonstrated the potential of MOS and QMB sensor arrays to discriminate samples according to species and their general aromatic profiles. Pennazza et al. demonstrated the ability of these systems to differentiate truffle species based on their volatile signatures, while Zampioglou and Kalomiros used an MOS-based electronic nose to distinguish species by their aromatic fingerprints [14,16]. More recently, electronic noses combined with chemometric analysis have been successfully used for the classification and grading of aromatic substances based on aroma intensity and quality. In this regard, Zampetti et al. demonstrated that MOS sensor arrays coupled with multivariate analysis allow for reliable classification and robust evaluation of aroma quality [17].

However, to date, the application of the analysis systems, including electronic noses, has focused on the classification and characterization of truffles in the laboratory, without yet constituting a direct detection tool in the field [3,14].

Traditional prospecting methods continue to be highly relevant. Trained dogs can locate ripe truffles with great precision, while pigs, although historically used, have limitations due to their tendency to consume the fungus [18]. The observation of “burns” or clearings, areas devoid of vegetation around the mycorrhizal tree, is another visual indicator of the presence of the fungus, although it does not guarantee the existence of mature carpophores [13,19,20]. There is a need to explore portable solutions adapted to field conditions. Optoelectronic noses, including a combination of optical and electrochemical sensors, offer a promising approach for in-field monitoring of volatile profiles due to their low cost, portability and the simplicity of data collection. They combine an array of sensors with advanced data analysis methods that generate response patterns on complex mixtures [21,22,23].

Thus, we hypothesize that an optoelectronic nose can be an appropriate approach to advance toward the detection of truffle buried in soil, and the accurate detection of *T. melanosporum* in real field conditions. To date, no optoelectronic systems specifically designed for this purpose have been reported. Therefore, the integration of chromofluorogenic sensors with electronic platforms represents an innovative way to improve the efficiency of identifying this species, providing a strategic tool for its detection.

## 2. Materials and Methods

### 2.1. Array Design

The detection system developed consisted of an optoelectronic device designed to identify *T. melanosporum* under controlled experimental conditions that simulate a real environment. The configuration integrates a set of optical sensors and an MQ-type electrochemical sensor. Table 1 lists the 10 sensor systems used, showing for each optical sensor the proportion of dye (mg) relative to the support (g). The set was defined after an initial screening by selecting those dyes of electrochemical systems that offered signal change. The operating principle is based on variations in color or electrical resistance in presence of the volatile pattern generated by the sample of interest.

The optical sensors were synthesized by impregnating three inorganic supports (UVM-7, silica, and alumina) with a variety of dyes with diverse chemical properties. The synthesis procedure involved dispersing the support in a dye solution in dichloromethane under stirring for 10 min, followed by a 24 h rest at room temperature to promote adsorption. Finally, the solvent was removed by rotary evaporation at reduced pressure, yielding optically active materials ready for use. They were arranged in ELISA-type plates with three replicates per sensor to ensure homogeneity in the response and reproducibility of the system. Also, MQ-136, an electrochemical sensor with a high response to compounds such as H_2_S, NH_3_, and SO_2_, was included in the array. The MQ-136 sensor (Hanwei Electronics Group Corporation, Zhengzhou, China) was connected directly to the analogue input of the ESP8266 microcontroller (Espressif Systems, Shanghai, China) (80 MHz CPU, 10-bit ADC, integrated WiFi, low power consumption). The analogue signal was acquired using the internal scaling voltage of the ESP8266′s ADC with an auxiliary processing unit Raspberry Pi 3 (1.2 GHz quad-core CPU, 1 GB RAM, integrated WiFi/Bluetooth). The software used was ESP8266 firmware and Raspberry Pi script for reading, transmitting, timestamping, and storing data in MongoDB and .txt files. A 30 min stabilization period was applied prior to data acquisition to minimize the baseline drift associated with the MQ sensor’s heating element. The signal was sampled at one-minute intervals, pre-processed by the ESP8266, and transmitted wirelessly to a Raspberry Pi 3 (Raspberry Pi Ltd., Cambridge, UK) for data storage on a microSD card and further processing.

### 2.2. Array Test and Data Acquisition

**Figure 1:**
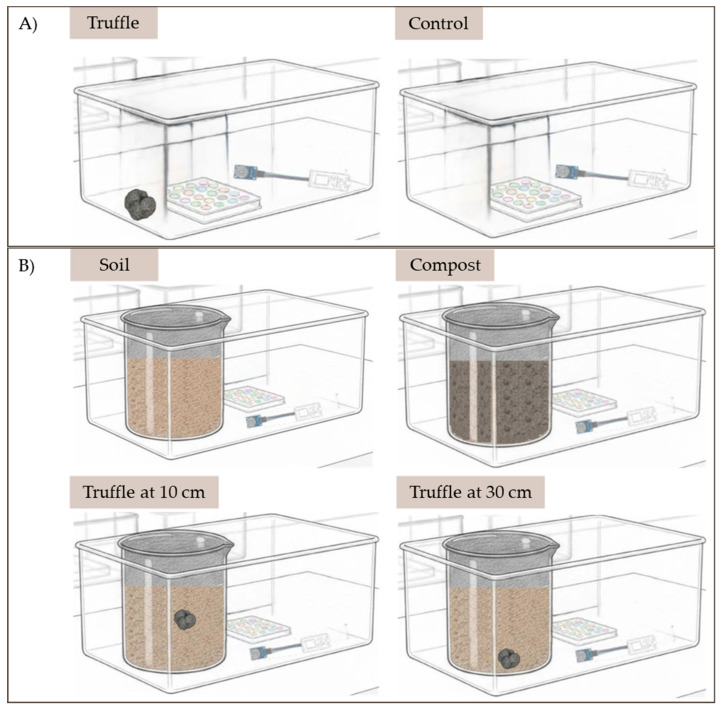
Experimental design followed in this study: (**A**) air/truffle tests; (**B**) test with buried truffle in presence of soil and compost.

In vitro tests were carried out using three closed 16-liter cavities (Figure 1): one as a control (air or soil only) and the other two containing the sample. Samples include truffle alone or truffle buried at 10 or 30 cm depth to simulate real in field conditions. Two tests were performed, in an initial air/truffle assays the sensors were exposed to the presence or absence of truffle. The assay was repeated on six different days, considered independent replicates. Once we confirmed the ability of the optoelectronic nose to identify the presence of truffle, a second series of assays were carried out by burying the truffle at diverse depths, with three independent experimental replicates per depth and eight independent replicates of soil without truffles. Also, a sample consisting of a soil mixed with compost (a strong generator of VOCs), a common material in agricultural soils, was used as control, with two independent replicates. This design made it possible to evaluate the discriminatory capacity of the optoelectronic nose against volatile compounds other than those of *T. melanosporum*, thus minimizing potential interference in detection. All experiments were conducted using fresh samples of *T. melanosporum* with post-harvest ages ranging from 2 to 8 days. Between experiments, the truffle samples were stored refrigerated (4 °C) to slow down metabolic activity and the evolution of volatiles. Before each measurement, the samples were equilibrated at room temperature to ensure reproducible headspace conditions. During the tests, the ELISA plates containing the optical sensors and the electrochemical sensor were placed inside the closed cavity for 7 h at room temperature. The electrochemical sensor remained operational for 2 h, recording signal variations every minute; measurements recorded during the first 20 min were discarded to minimize drift effects. Before each test, MQ-136 underwent a stabilization process that included preheating and a 30 min adaptation period to avoid initial signal.

Images of the chromofluorogenic array were acquired before and after the experiment using a Canon EOS R50 camera (Canon Inc., Tokyo, Japan). To ensure reproducibility color change images were taken in a light box with high-intensity LED plates, and fluorescence images were collected using ultraviolet light at 254 nm and 365 nm. Data acquisition from the electrochemical sensors was carried out via the analogue pins of an ESP8266 microcontroller, recording the voltage signal generated, discarding the initial readings to avoid biases associated with drift. The analogue signal was sampled at one-minute intervals and then normalized by subtracting the reference value obtained in clean air (R_0_) from each resistance measurement (R_1_). Chromofluorogenic data from the optical and fluorescence sensors were extracted using custom software developed in Python 3.11, which allowed for the RGB coordinates of each optical sensor to be retrieved automatically. Based on these values, the ΔE parameter (Equation (1)) was calculated as a quantitative indicator of the color difference between unexposed plates (control, R_0_) and plates after exposure to volatile compounds (R_1_). This metric, based on the overall difference in color space, allows for the integrated quantification of color variations induced by VOCs, offering high sensitivity and the advantage of using a single comparable parameter for all detection materials, regardless of their color.

(1) $$\Delta E=\sqrt{{\left(R_{1}-R_{0}\right)}^{2}+{\left(G_{1}-G_{0}\right)}^{2}+{\left(B_{1}-B_{0}\right)}^{2}}$$

### 2.3. Data Analysis

The signals from the electrochemical and optical sensors were manually integrated into a structured database to perform the corresponding multivariate analyses. Before constructing the predictive models, a pre-processing step was applied using auto-scaling, with the aim of normalizing the variables and ensuring their comparability. Principal component analysis (PCA) was performed with the IBM SPSS Statistics software (Version 28.0.1.1). PCA was applied to the input data using the Varimax rotation method, which maximizes the variance of the squared loadings within each component, facilitating a clearer interpretation of the results. Partial least squares discriminant analysis (PLS-DA) was performed using the R programming environment and the mdatools library. To assess the reliability and robustness of the models, the Leave One Out (LOOCV) cross-validation technique was used, which allowed for the predictive performance to be estimated and ensured adequate generalization capacity. Finally, an artificial neural network (ANN) adjusted to the experimental data was implemented using the NeuralNet package in R. The model architecture was tested with 2 to 6 neurons in the hidden layer, depending on the optimal configuration for predicting each group. For the ANN model evaluation, two-thirds of the data were used for calibration, while the remaining third was reserved for validation.

## 3. Results and Discussion

### 3.1. Implementation of an Optoelectronic System for the Identification of T. melanosporum

To overcome the limitations of traditional truffle prospecting methods, such as the use of animals [14] or identification based on aromatic profiles in the laboratory [3,7], a portable optoelectronic system was developed for the detection of *T. melanosporum* under controlled conditions that simulate the underground environment. This device integrates nine optical sensors and one electrochemical sensor, selected for their potential ability to generate color, fluorescence intensity, or resistance variations. The optical sensors include commercial dyes, which facilitate scaling up and cost reduction. The dyes were chosen for their high molar extinction coefficient, which minimized the amount of material needed without compromising detection efficiency. In addition, the sensors were deposited on three inorganic supports—UVM-7 mesoporous silica, aluminum oxide, and conventional silica—whose surface properties and acid–base character modulate the response of the dyes. The bimodal porous structure of UVM-7 facilitates the diffusion of volatile compounds, optimizing the sensitivity of the system [24,25]. Electrochemical sensors, although not completely selective, provide complementary information on the presence of certain gases, contributing to a more robust detection profile. In this context, the recent literature has demonstrated that electronic noses can discriminate not only the identity of a sample, but also differences in the intensity or degree of aromatic expression, based on the global response of the sensory array. This capability is particularly relevant for complex biological matrices such as truffles, where variations in volatile emission may be associated with maturity, environmental conditions, or soil interactions [12,13,14]. In line with this background, the classification performance obtained in this study is consistent with previous work using electronic nose systems for aroma classification, in which metal oxide sensor (MOS) arrays, combined with multivariate models, have demonstrated high robustness and sensitivity to volatile profiles [17].

The design of the optoelectronic system is based on the group’s previous experience in developing tools for detecting volatile compounds in agro-environmental settings. It is closely related to the system developed for the detection of Cotonet de les Valls in citrus fruits [23], which combined colorimetric and electrochemical optical sensors to identify specific volatile signals from the pest. Both systems share the same detection philosophy, based on the generation of multivariate chemical fingerprints obtained from hybrid sensory matrices. However, the device proposed for the detection of *T. melanosporum* introduces a significant improvement by incorporating fluorescent response sensors, which extend the spectral range of detection and improve the ability to discriminate against environmental interference. This diversification in response mechanisms contributes to greater sensitivity and robustness of the system, especially in complex conditions such as those associated with the presence of soil or organic matter. The integration of both types of sensors allows for the obtention of chromofluorogenic and electrochemical fingerprints, offering a potential non-invasive and robust method for the detection of *T. melanosporum*.

It is important to note that, in this study, no prior microextraction or passive preconcentration steps were used, as the main objective was to evaluate the intrinsic ability of the optoelectronic system to detect the characteristic volatile signature of *T. melanosporum* through direct sampling of the headspace. This approach allows for the performance of the device to be evaluated under simplified and reproducible conditions, providing a solid basis for future improvements to the system without introducing additional complexity that could mask the actual contribution of the sensory matrix.

### 3.2. Sensory Response Profile to Volatiles

**Figure 2:**
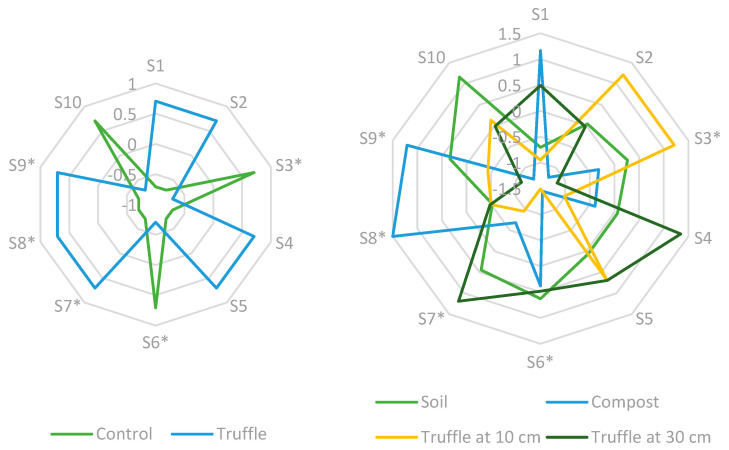
Response of the sensors recorded during the initial air/truffle assays (control and truffle) and in the second series of assays (soil, truffle at 10 cm, truffle at 30 cm, and compost).

The optoelectronic system was evaluated to determine its ability to recognize VOC patterns emitted by *T. melanosporum* and subsequently validate its performance through in vitro testing. Two complementary graphical representations were generated (Figure 2). The first shows the radial profiles corresponding to the initial air/truffle assays, explicitly comparing the responses with truffles against those of the control without truffles; this graph illustrates the presence/absence of response to the direct volatile stimulus. The second compares the responses obtained in the second series of assays under four conditions: soil only (control), truffle buried at 10 cm, truffle buried at 30 cm, and soil with compost on the surface. This second graph allows for the effect of the soil matrix and organic matter on the sensory pattern to be evaluated.

A comparison of the two graphs (Figure 1) reveals consistent differences in sensory profiles: the set of sensors presents a distinguishable fingerprint when truffles are present and modified patterns in the presence of compost or soil alone. These naked eye pattern differences support the ability of the optoelectronic nose to both detect the presence of *T. melanosporum* and differentiate it from other sources of VOCs (compost), confirming the suitability of the approach for prospecting and management applications in truffle farms.

Although the array response is due to the interaction with the full set of volatiles present in the headspace, based on the potential chemistry of the colorants and their possible interactions with truffle volatiles, as reported in the literature [2,26], it is possible to propose specific response mechanisms for each sensor. For example, Rhodamine 6G (S1) could interact reversibly with aldehydes or amines present in truffles, such as hexanal or 2,3-butanedione. The pH indicators m-Cresol purple (S2) and Cresol red (S4) could respond to variations in acidity or basicity caused by short-chain fatty acids or volatile nitrogen compounds generated by truffles or their microbiota. Basic and anionic dyes, such as Safranine O (S7) and Congo red (S3), could interact with phenolic acids or minor polar compounds, altering their spectral absorption. Finally, reactive fluorophores such as Fluorescein 5(6)-isothiocyanate (S8) could form covalent bonds with volatile amines, generating changes in the fluorescence signal. These hypotheses on the correlation between the optical signals obtained and the chemical composition of truffle volatiles provide a basis for interpreting the sensor’s response patterns.

**Figure 3:**
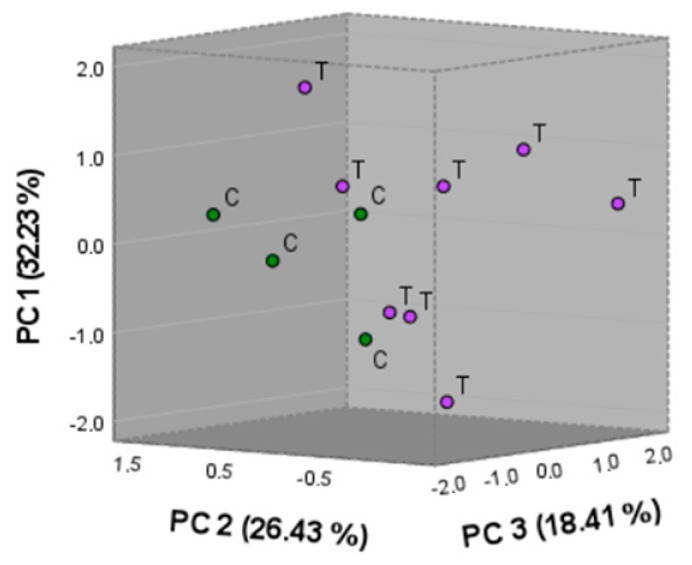
PCA projection of the responses of the optoelectronic nose obtained in the initial air/truffle assays, comparing samples with (T) and without (C) *T. melanosporum*.

Principal component analysis (PCA) was applied to simplify the complexity of the data generated by the chromofluorogenic and electrochemical sensors. This statistical tool allowed for the responses to be projected into a multivariate space defined by the first three principal components (PC1, PC2, and PC3), providing a clearer visualization of the relationships between the variables. In the initial air/truffle assays (Figure 3), PCA showed a clear separation between samples with truffles (group ‘T’) and control samples without truffles (group ‘C’).

The PCA of the first three PCs of the second series of assays with four experimental conditions—truffle at a depth of 10 cm (T1), truffle at 30 cm (T2), control soil (S), and compost (C)—does not place each sample in defined groups. This limited separation suggests that the first three principal components do not capture the total explanatory variability of the system, so complete discrimination between these groups probably requires the consideration of additional components. From a functional point of view, this observation could be explained by the attenuation of the VOC signal emitted by the truffle when it is underground, which reduces the contrast with the bare soil profile. This behavior is consistent with Fickian diffusion models in porous media, where gas transport through the soil is governed by concentration gradients and is strongly affected by soil porosity, tortuosity, and adsorption processes [27]. Even so, the observed trend confirms that the system can detect latent differences, although not fully resolved in the three-dimensional space analyzed, which highlights both the possibilities and the limitations of the approach in conditions of high complexity with mass-transfer/sensitivity issues.

This analysis highlights the influence of the environment on the response of chemical sensors. Although the sensor array performed optimally under controlled conditions, its overall effectiveness decreased under simulated field environments because of matrix complexity and reduced VOC diffusion. This behavior reveals that the simple exposition to truffle can be not representative, and that the in vitro test including soil is essential to identify the most robust and efficient sensors, ensuring their applicability in the detection of *T. melanosporum*.

### 3.3. Identification of the Presence of Truffle Using PLS-DA

To evaluate the classification capacity of the *T. melanosporum* detection system, PLS-DA models were developed using the signal from the set of 10 sensors. The use of PLS-DA is particularly appropriate in this context, as it is a method designed for categorical classification problems, allowing both binary and multi-class scenarios to be addressed. It was applied to the data obtained in the initial air/truffle tests whose objective was to discriminate between the presence (T) and absence (C) of truffles. Subsequently, the method was applied to the classification between the four conditions studied in the second assay: soil without truffles (S), truffles buried at 10 cm (T1), truffles buried at 30 cm (T2), and compost (C). The model performance indicators (TPR, TNR, precision, F-score, and accuracy) are presented in Table 2 and Table 3.

**Figure 4:**
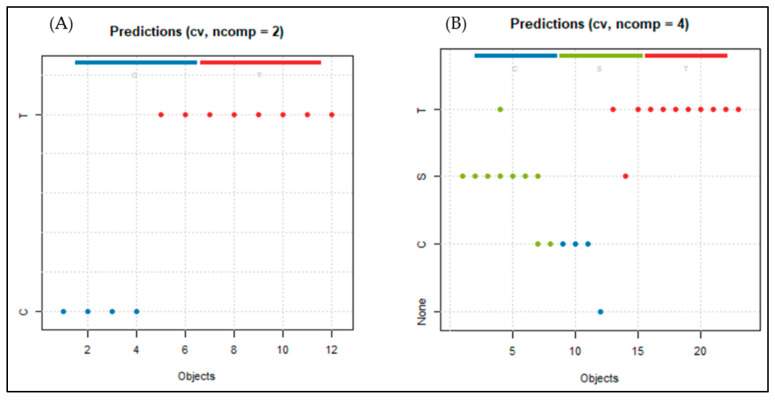
Predictions in validation of the PLS-DA model for (**A**) air/truffle tests; (**B**) test with buried truffle in presence of soil and compost.

In the proof-of-concept tests (presence/absence of truffles), following the results of PCA, cross-validation showed an accuracy of 1.000, as well as maximum values for the rest of the model evaluation metrics (Table 2), confirming its ability to achieve a perfect separation between the two classes. Similarly, Figure 4A shows that the model correctly classifies all samples, supporting its high performance under these initial experimental conditions. These results demonstrate the system’s effectiveness in identifying truffles in initial air/truffle tests, while encouraging further testing in more complex environments (presence of soil and organic matter) to assess its robustness against environmental variations. 

In the second series of tests carried out in the presence of soil (Table 3), the PLS-DA model showed adequate overall performance, with accuracies of 0.913 in most classes. Given that truffle identification is the main objective of the system, classes T1 and T2 are particularly relevant. In this regard, the identification of truffles (T1 and T2) showed accuracy (0.500–0.833) and F-score (0.615–0.833) values, together with a TPR of round 0.800, indicating a moderate identification ability that could be dependent on truffle depth. Analysis of the other classes reveals that S is the most stable and homogeneous class in terms of its metrics, demonstrating adequate discrimination of soil as an interfering matrix. Class C, despite having a perfect TPR, showed moderate precision (0.667) associated with certain false positives. Some S samples were misclassified as truffles and one truffle sample was assigned to S, contributing to the decrease in performance, especially in T1. Taken together, these results show that the system has difficulties differentiating among truffles at diverse depths, and presence of soil introduces greater complexity into the system’s response pattern. Overall, these results support the idea that the array/model could be a viable approach detect buried truffles, although it has limitations given the complexity of real matrices and the variety of classes.

Thus, in order to simplify the system, buried truffles were merged into a single category (T). This approach significantly improves the consistency of the model (Table 4, Figure 4B), achieving high and balanced values across all metrics (TPR, precision, and F-score = 0.909; accuracy = 0.913), indicating much more stable discrimination than when depths were evaluated separately. Class S (absence of truffle) maintains an equally robust and consistent performance confirming a low tendency to offer false positives. In contrast, class C shows the most limited performance, with an accuracy of 0.600 and a TPR of 0.750, reflecting a certain tendency towards false positives for this class. The performance of the system is illustrated in Figure 4B, which shows the discrepancies in the classification of the samples. Specifically, one soil sample was incorrectly assigned as truffle, one sample from the truffle class was classified as S, and two samples from the S class were assigned to the C class, highlighting the specific limitations of the model in discriminating between these classes. These improvements reflect that the model focuses is able to differentiate between samples containing truffles and those that do not. This simplified approach provides a more robust and reliable response for practical field applications, where detecting the presence of truffles is more relevant than estimating their depth.

**Figure 5:**
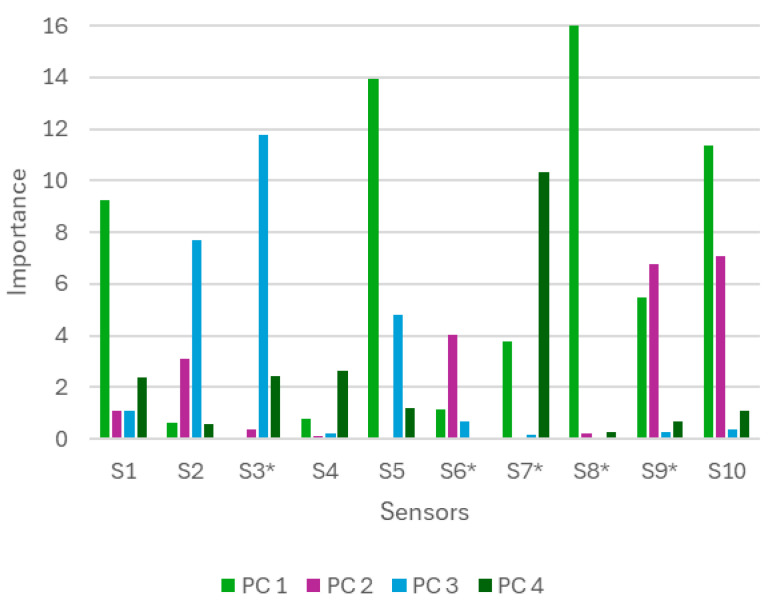
Contribution of each sensor to the main components of the PLS-DA.

In this context, the analysis of the loads associated with the principal components provides additional information on the relevance of each sensor in the PLS-DA model discrimination. Figure 5 shows that PC1 is dominated by S1, S5, S8*, and S10, which could be related to the detection of reducing sulfurous compounds, such as dimethyl sulfide, characteristic of truffles. The colorimetric and fluorogenic sensors could respond to spectral or fluorescence changes due to interactions with aldehydes, amines, or sulfhydryl groups, while the MQ-136 is sensitive to sulfurous gases conductimetrically. PC2, dominated by S6*, S9*, and S10, could reflect variations in volatile nitrogen compounds or acidity, such as amines or ammoniacal compounds, detected by fluorescence and conductivity. PC3, with S2 and S3*, could be associated with phenolic compounds or organic acids that affect the protonation of dyes, and PC4, defined by S7*, could capture minor volatiles or more complex interactions, sensitive to changes in polarity or acidity. In general, the system’s discrimination is based on the complementarity of colorimetric, fluorescent and electrochemical sensors, each sensitive to specific chemical classes of truffle volatiles, allowing subtle variations to be captured under different experimental conditions.

### 3.4. Classification Capability of Neural Networks for the Detection of T. melanosporum

The use of artificial neural networks (ANNs) is another common approach to multivariate data modelling. While both ANNs and PLS-DA can be used to address classification problems, they differ in their architecture and predictive power. ANNs offer the advantage of capturing non-linear relationships and complex patterns in data. In this study, a classification neural network was implemented to model the relationship between sensor responses and the different experimental classes considered. The classification results obtained from neural networks applied to initial air/truffle tests showed moderate discrimination between classes (Table 5). The prediction matrix indicated correct identification of all truffle samples (sensitivity = 1.00), while the non-truffle class (C) had a higher error rate, with 50% accuracy. Overall accuracy reached 75% (95% CI: 0.19–0.99), with a specificity of 0.667 and a balanced accuracy of 0.833. However, the kappa value (0.50) suggests moderate agreement between the predicted and actual classification, reflecting acceptable but still improvable initial performance in differentiating between the two classes.

The neural network model applied to the second series of tests (Table 6), which included four classification groups (C, S, T1, and T2), showed moderate overall performance, with an accuracy of 62.5% (95% CI: 0.24–0.91) and a kappa coefficient of 0.489. Despite this variability, the system showed a remarkable ability to identify the presence of buried truffles, especially in condition T2, where it achieved a sensitivity of 1.00 and a balanced accuracy of 0.833. Class T1 also performed acceptably (balanced accuracy = 0.750), indicating that the neural network recognizes samples containing truffles more reliably than interfering matrices. In contrast, class S (soil) had the highest confusion rate, with 33% of samples misclassified as T2, which limits the specificity of the model for certain types of interfering matrix. Nevertheless, the discrimination between truffle and non-truffle remains at promising levels, demonstrating the system’s potential for automatic detection of buried truffles.

In the three-group classification, as in the PLS-DA, the model showed significantly better performance than in the four-class scenario (Table 7). Overall accuracy reached 87.5% (95% CI: 0.47–0.99), along with a kappa coefficient of 0.784, indicating a high level of agreement between the prediction and the actual classification. All compost and soil samples were correctly identified (sensitivity = 1.00), while class T1 also performed robustly, with a sensitivity of 0.80 and a balanced accuracy of 0.90. These results confirm that, by combining the two truffle depths into a single category, the model becomes more stable and clearly improves its ability to distinguish truffle samples from other interfering matrices.

**Figure 6:**
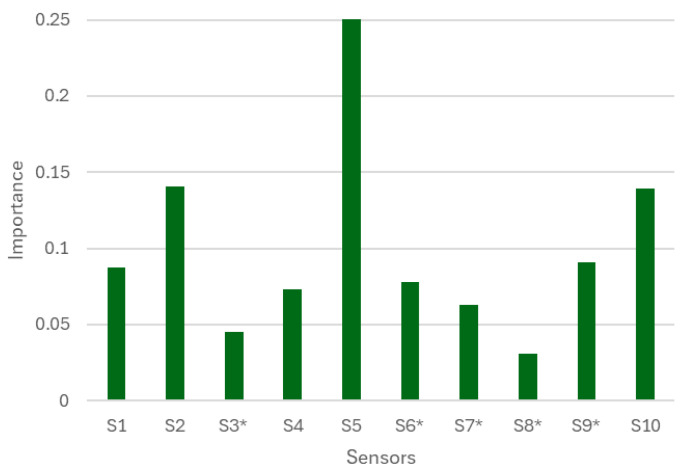
Contribution of the sensors to the artificial neural network model according to Garson’s importance values.

In relation to the contribution of each sensor to the model, Garson’s importance values (Figure 6) indicate that sensors S2, S5, and S10 have the greatest weight in the classification. These are two colorimetric sensors supported by UVM-7 and silica, respectively, together with the electrochemical sensor. In addition, each of them has also played an important role in two of the main components of the PLS-DA model, reinforcing their relevance in both supervised analysis and the neural network.

In general, the comparison between the two methods shows that PLS-DA works more stably and reliably to detect *T. melanosporum* in all the conditions studied. Although neural networks are also capable of identifying truffles, their performance is more irregular and is more affected by interfering matrices, which generates more errors in scenarios with several classes. In contrast, PLS-DA maintains more balanced and consistent results, and performs even better when truffles are considered as a single group. 

This behavior can be explained by the quasi-linear and attenuated nature of the sensor response in soil conditions, where the diffusion of volatile compounds and interactions with the matrix generate relatively stable multivariate trends. PLS-DA can effectively capture these covariance-based relationships, while neural networks tend to overfit noise or specific features of small and variable datasets, limiting their robustness. Taken together, these results suggest that PLS-DA is the most reliable strategy for detecting truffles under the controlled conditions evaluated in this study. However, these experimental conditions do not fully replicate the complexity of field scenarios, where factors such as VOC diffusion and environmental heterogeneity could influence the system’s performance.

### 3.5. Influence of Sensor Type on the Discriminatory Capacity of the Models

To further evaluate the relevance of the combination of diverse type of sensors on the system’s performance, the models generated using subsets of sensors were analyzed and compared with the complete multi-source system (Table 8 and Table 9). The results obtained with PLS-DA and neural networks show that the simultaneous integration of colorimetric, fluorogenic, and electrochemical signals is crucial to maintaining optimal performance.

In the case of PLS-DA (Table 8), the removal of the MQ electrochemical sensor reduced the quality of the model, with TPR values ranging from 0.5 to 0.727 and accuracies ≤ 0.826 in classes C, S, and T. Models built exclusively with colorimetric or fluorescent sensors showed even greater deterioration: in some cases, the TPR was zero and the accuracies did not exceed 0.87. Overall, the results show that no partial group of sensors is capable of reproducing the performance achieved when all signal sources are combined.

A similar trend was observed in the neural network models (Table 9). Without the MQ sensor, classes C and S had a sensitivity of 0.000 and a balanced accuracy of 0.500, indicating random behavior. Meanwhile, the exclusive use of colorimetric sensors resulted in even worse performance (sensitivities of 0.000 for C and S and an overall accuracy of 0.375). Fluorescent sensors show a slight improvement in class S (sensitivity 0.667, specificity 0.600, balanced accuracy 0.633), but classes C and T remain at values close to random (0.500). In contrast, when the neural network incorporated the three signal sources, high sensitivity and accuracy values were obtained, meaning that effective discrimination is only achieved through multi-source integration.

Taken together, these results show that the multi-source nature of the system is essential for maximizing the discriminatory capacity of both linear and non-linear models. The complementary combination of colorimetric, fluorogenic, and electrochemical sensors allows for the capture of a greater diversity of volatile compounds and chemical patterns, which translates into superior performance and greater robustness of the system in the face of complex matrices.

## 4. Conclusions

This study evaluates the applicability of an optoelectronic nose for the non-invasive detection of *Tuber melanosporum* under controlled conditions with outstanding performance in initial presence/absence tests (accuracy = 1.000) and robust detection of buried truffles, especially at a depth of 30 cm (TPR = 0.833; accuracy = 0.913). Reclassifying truffles into a single category improved the consistency of the PLS-DA model (TPR, precision, and F-score = 0.909), while neural networks, despite their greater variability, achieved a sensitivity of 1.000 for the T2 group and an accuracy of 0.875 in the three-class scenario. The system requires the combination of colorimetric, fluorogenic, and electrochemical sensors. In addition to its discriminatory effectiveness under lab conditions, the device has clear advantages such as ease of use, non-invasiveness, and elimination of the need for animals. However, the test and models were obtained in a 16L plastic box; thus, further studies in field would be necessary for its validation. Taken together, these results support the potential of optoelectronic noses as reliable, efficient, and transferable tools for the early detection of *T. melanosporum*, with promising applications in both research and the agronomic management of truffle plantations.

## Figures and Tables

**Table 1:** Set of sensors chosen for the optoelectronic nose.

		Dye	Support	mg dye/g Support
Optical sensors	S1	Rhodamine 6G	UVM-7	1.00
	S2	m-Cresol purple	UVM-7	0.95
	S3*	Congo red	UVM-7	1.19
	S4	Cresol red	Alumina	0.89
	S5	Victoria blue B	Silica	0.28
	S6*	Acridine orange	Silica	0.29
	S7*	Safranine 0	Silica	0.31
	S8*	Fluorescein 5(6)-isothiocyanate	Silica	0.46
	S9*	Dichlorofluorescein	Silica	0.46
Electrochemical sensors	S10	MQ136		

* Indicates that the reported signal corresponds to a change in fluorescence.

**Table 2:** Summary of the PLS-DA, including cross-validation results for initial air/truffle assays (samples with truffle (T) and without truffle (C)).

Class	TPR	TNR	Precision	F-score	Accuracy (%)
C	1.000	1.000	1.000	1.000	100.0
T	1.000	1.000	1.000	1.000	100.0

**Table 3:** Summary of the PLS-DA, including cross-validation results for trials with truffles buried at different depths (soil (S), truffles at 10 cm (T1), truffles at 30 cm (T2), and compost (C)).

Class	TPR	TNR	Precision	F-score	Accuracy (%)
C	1.000	0.895	0.667	0.800	91.3
S	0.875	0.933	0.875	0.875	91.3
T1	0.800	0.778	0.500	0.615	78.3
T2	0.833	0.941	0.833	0.833	91.3

**Table 4:** Summary of the PLS-DA, including cross-validation results for classification into three classes (truffle, soil, and compost).

Class	TPR	TNR	Precision	F-score	Accuracy (%)
C	0.750	0.895	0.600	0.667	87.0
S	0.875	0.933	0.875	0.875	91.3
T	0.909	0.917	0.909	0.909	91.3

**Table 5:** Performance of the artificial neural network model for the initial air/truffle trials: global metrics.

Accuracy (%)	95% CI	*p*-Value [Acc > NIR]	Kappa	Sensitivity (Recall)	Specificity	Balanced Accuracy
75.0	(0.1941, 0.9937)	0.7383	0.5	1.000	0.667	0.833

**Table 6:** Performance of the artificial neural network model for the second series of assays: global metrics and metrics by class.

**Accuracy (%)**	**95% CI**	***p*** **-Value [Acc > NIR]**	**Kappa**
62.5	(0.2449, 0.9148)	0.1374	0.4894
**By Class**	**Sensitivity (Recall)**	**Specificity**	**Balanced Accuracy (%)**
C	1.000	1.000	100.0
S	0.333	0.800	56.7
T1	0.500	1.000	75.0
T2	1.000	0.667	83.3

**Table 7:** Performance of the artificial neural network model for the second series of assays with 3 classes: global metrics and metrics by class.

**Accuracy (%)**	**95% CI**	***p*** **-Value [Acc > NIR]**	**Kappa**
87.5	(0.4735, 0.9968)	0.135	0.7838
**By Class**	**Sensitivity (Recall)**	**Specificity**	**Balanced Accuracy (%)**
C	1.000	1.000	100.0
S	1.000	0.833	91.7
T	0.800	1.000	90.0

**Table 8:** Evaluation of the performance of the PLS-DA models according to the sensor subset: without the MQ sensor, only colorimetric sensors, and only fluorescent sensors.

	Class	TPR	TNR	Precision	F-score	Accuracy
Without MQ	C	0.500	0.895	0.500	0.500	0.826
	S	0.500	0.667	0.444	0.471	0.609
	T	0.727	0.833	0.800	0.762	0.783
Colorimetric	C	0.500	0.947	0.667	0.571	0.870
	S	0.000	0.733	0.000	0.000	0.478
	T	0.455	0.667	0.556	0.500	0.565
Fluorescent	C	0.250	0.895	0.333	0.286	0.783
	S	0.250	0.733	0.333	0.286	0.565
	T	0.636	0.667	0.636	0.636	0.652

**Table 9:** Evaluation of the performance of the artificial neural network based on the subset of sensors.

	**Class**	**Sensitivity (Recall)**	**Specificity**	**Balanced Accuracy**
Without MQ	C	0.000	1.000	0.500
	S	0.000	1.000	0.500
	T	1.000	0.000	0.500
Colorimetric	C	0.000	1.000	0.500
	S	0.000	0.667	0.333
	T	0.600	0.000	0.300
Fluorescent	C	0.000	1.000	0.500
	S	0.667	0.600	0.633
	T	0.500	0.500	0.500
**Global**	**Accuracy**	**95% CI**	***p*-Value [Acc > NIR]**	**Kappa**
Without MQ	0.500	(0.157, 0.843)	0.6367	0.000
Colorimetric	0.375	(0.085, 0.755)	0.9640	−0.333
Fluorescent	0.500	(0.157, 0.843)	0.6367	0.111

## Data Availability

The original contributions presented in this study are included in the article. Further inquiries can be directed to the corresponding author.

## Abbreviations

The following abbreviations are used in this manuscript:

PLS-DA	Partial Least Squares Discriminant Analysis
ANN	Artificial Neural Network
VOCs	Volatile Organic Compounds
GC-MS	Gas Chromatography coupled with Mass Spectrometry
HS-SPME	Headspace Solid-Phase Microextraction
MOS	Sensors Metal Oxides
PCA	Principal Component Analysis
LOOCV	Leave One Out Cross-Validation
PC	Principal Component
CI	Confidence Interval
TPR	True Positive Rate
TNR	True Negative Rate
